# Impact of yellow fever vaccine on patients with psoriasis: preliminary results^☆^^☆☆^

**DOI:** 10.1016/j.abd.2018.11.001

**Published:** 2019-10-26

**Authors:** Mayara Hamilko de Barros, João Carlos Regazzi Avelleira, Kleiser Aparecida Pereira Mendes

**Affiliations:** a Psoriasis Outpatient Clinic, Instituto de Dermatologia Professor Rubem David Azulay, Santa Casa de Misericórdia do Rio de Janeiro, Rio de Janeiro, RJ, Brazil; b Department of Allergies and Dermatological Immunology, Setor de Alergia e Imunologia Dermatológica, Instituto de Dermatologia Professor Rubem David Azulay, Santa Casa de Misericórdia do Rio de Janeiro, Rio de Janeiro, RJ, Brazil

*Dear Editor*,

Brazil is currently experiencing adversity with new occurrences and the expansion of sylvatic yellow fever. The Brazilian Ministry of Health reported 1286 human cases of the disease between July 2017 and February 2018. The data shows 353 confirmed cases, 98 deaths and the Southeast region was the most affected.^1^

The epidemiological importance of the disease comes from its high potential for dissemination, the risk of reorganization, and its clinical severity (with a fatality rate of up to 50% among severe cases).^1^

The most effective strategy for combating yellow fever is based on extensive vaccination of the population in risk areas, after considering the impracticality of eliminating vector mosquitoes and the absence of treatment for the disease. In 2016, the epidemic in the Democratic Republic of Congo was stopped with fractional doses and large-scale vaccination.

The vaccine is considered to be effective and safe, although it is a live attenuated virus. However, serious and even fatal adverse events have been reported.^2^ Among the contraindications of the vaccine we find autoimmune and immunosuppressive diseases, as well as those who use immunosuppressive/immunomodulatory therapies.^3^

Psoriasis is genetic-based inflammatory disease, with the participation of innate (auto-inflammatory) and acquired (autoimmune) immunity. It has a prevalence of 1–2% in the world population, and many patients use potentially immunosuppressive drugs, such as methotrexate, cyclosporine, and biological agents (anti-TNF, antiTh17, anti-IL17). According to these factors, the recommendation for immunization and the risk of serious adverse effects should be evaluated and compared with the risk of contracting the disease in patients living in areas where vaccination is indicated.

Considering that many vaccines can trigger or worsen autoimmune diseases, what would be the impact of the yellow fever vaccine on the clinical evolution of psoriasis?

Here we report a cross-sectional retrospective study reviewing the records of 63 patients with psoriasis who received the vaccine against yellow fever inadvertently and independently of medical indication in 2017 in the Brazilian states of Rio de Janeiro, São Paulo, Espírito Santo, Minas Gerais, and Bahia.

In this study (63 patients), 52.3% were women, 84.1% were from the state of Rio de Janeiro, and the average age was 46.4 years (SD ± 17.42) (Table 1). Regarding treatment, 1.5% were untreated, 34.9% received topical treatment exclusively, and 63.4% used systemic treatment (methotrexate 22.5%, acitretin 10%, etanercept 27.5%, adalimumab 25%, ustekinumab 10%, and infliximab 5%) (Fig. 1).

**Table 1:** Epidemiological description of the sample (*n* = 63).

	Number of patients (%)
*Gender*	
Female	33 (52.3%)
Male	30 (47.6%)

*Age*	
≤15 years old	6 (9.5%)
16–30 years old	4 (6.3%)
31–60 years old	41 (65%)
>60 years old	12 (19%)

*Origin*	
Rio de Janeiro	53 (84%)
Espírito Santo	4 (6.3%)
Minas Gerais	4 (5%)
São Paulo	1 (1.5%)
Bahia	1 (1.5%)

**Figure 1:**
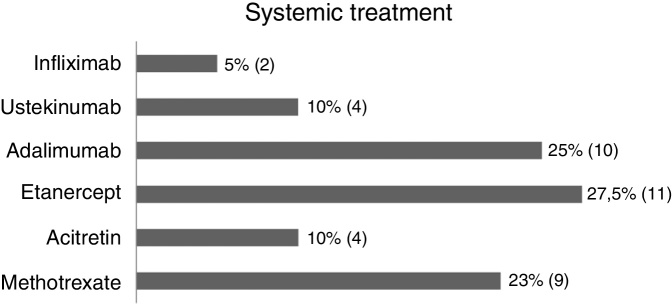
Systemic treatment for psoriasis at the time of vaccination against yellow fever (medication *vs.* percentage/number of patients).

Sixty-one patients received the vaccine for the first time and had no adverse effects. Two patients reported post-vaccine reaction: headaches (using acitretin) and vasculitis (using topical). There was no change in the clinical course of post-vaccination psoriasis in 93.6% of the patients, 4.7% reported improvement (etanercept, adalimumab, topical), and 1.5% reported worsening of the lesions (acitretin) (Fig. 2).

**Figure 2:**
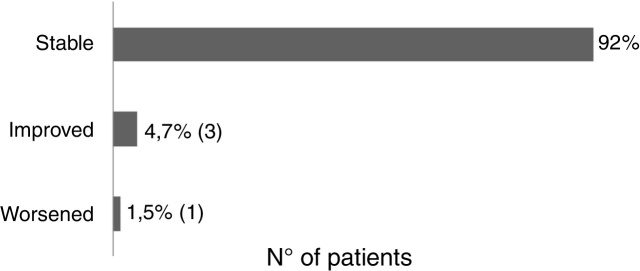
Evolution of psoriasis after yellow fever vaccine.

In literature, we find articles that correlate rheumatic diseases and yellow fever vaccine^3^, ^4^; however, there are no studies to date evaluating its impact in patients with psoriasis.

In 2009, Mota et al. evaluated 70 patients with rheumatic diseases using immunosuppressants that were inadvertently vaccinated. They found no major adverse effects, although the vaccine was contraindicated because of the increasing risk of acute viscerotropic disease in this group.^4^ Some of the other factors implicated in the etiology of this adverse effect are patients with age of 60 years or greater, precursors of thymectomy, and autoimmune diseases.^3^ It is also believed that adverse effects occur due to dysfunction of the signaling between the innate and adaptive systems.^2^

The potential association of vaccination in the induction or exacerbation of autoimmune diseases has been questioned, although it is known that autoimmune reactions to vaccines can occur rarely in predisposed individuals, most likely by molecular mimicry.^5^

The pathophysiology of psoriasis involves activation of dendritic cells, neutrophils, and T cells, mainly CD8+, with consequent production of IL-8, TNF-α, and IL-17. Psoriasis treatments are based on immunosuppression/immunomodulation (methotrexate, anti-TNF-α, and anti-IL-17) to reduce or neutralize the production of cytokines. However, these same cytokines are also involved in protecting the individual against intracellular pathogens such as viruses and mycobacteria. In this way, such immunosuppression could trigger vaccine infection by hindering adequate response to the attenuated virus.

Adverse events in our sample were mild and rare, and there was no evidence of severe manifestation, hospitalization, or death in patients with psoriasis. Nor was there any change in the clinical course of post vaccination psoriasis, regardless of treatment (with or without immunosuppressive drugs).

Sample size is obviously one of the limitations for more definitive knowledge of the prevalence of adverse effects and the behavior of the disease after vaccination, in order to establish guidelines or protocols for the guidance of health professionals and patients; however, this represents a record in progress that is being enlarged every month by the authors.

## Financial support

None declared.

## Author's contributions

Mayara Hamilko de Barros: Statistical analysis; approval of the final version of the manuscript; conception and planning of the study; elaboration and writing of the manuscript; obtaining, analyzing and interpreting the data; effective participation in research orientation; intellectual participation in propaedeutic and/or therapeutic conduct of the cases studied; critical review of the literature; critical review of the manuscript.

João Carlos Regazzi Avelleira: Statistical analysis; approval of the final version of the manuscript; conception and planning of the study; elaboration and writing of the manuscript; obtaining, analyzing and interpreting the data; effective participation in research orientation; intellectual participation in propaedeutic and/or therapeutic conduct of the cases studied; critical review of the literature; critical review of the manuscript.

Kleiser Aparecida Pereira Mendes: Approval of the final version of the manuscript; elaboration and writing of the manuscript; critical review of the literature.

## Conflicts of interest

None declared.
